# Coronavirus in Continuous Flux: From SARS‐CoV to SARS‐CoV‐2

**DOI:** 10.1002/advs.202001474

**Published:** 2020-08-16

**Authors:** Yetian Dong, Tong Dai, Jun Liu, Long Zhang, Fangfang Zhou

**Affiliations:** ^1^ Institutes of Biology and Medical Sciences Soochow University Suzhou 215123 P. R. China; ^2^ Life Sciences Institute and Innovation Center for Cell Signaling Network Hangzhou Zhejiang 310058 P. R. China; ^3^ Pinghu Food and Drug Inspection Center Pinghu Zhejiang 314200 P. R. China

**Keywords:** biology, clinical symptoms, immune responses, SARS‐CoV‐2, therapeutic treatment

## Abstract

The world is currently experiencing a global pandemic caused by a novel coronavirus, severe acute respiratory syndrome coronavirus 2 (SARS‐CoV‐2), which causes severe respiratory disease similar to SARS. Previous studies have suggested that SARS‐CoV‐2 shares 79% and 96% sequence identity to SARS‐CoV and to bat coronavirus RaTG13, respectively, at the whole‐genome level. Furthermore, a series of studies have shown that SARS‐CoV‐2 induces clusters of severe respiratory illnesses (i.e., pneumonia, acute lung injury, acute respiratory distress syndrome) resembling SARS‐CoV. Moreover, the pathological syndrome may, in part, be caused by cytokine storms and dysregulated immune responses. Thus, in this work the recent literature surrounding the biology, clinical manifestations, and immunology of SARS‐CoV‐2 is summarized, with the aim of aiding prevention, diagnosis, and treatment for SARS‐CoV‐2 infection.

## Graphical abstract

Severe acute respiratory syndrome coronavirus 2 (SARS‐CoV‐2) has posed a serious threat to public health. Since the outbreak, numerous attempts have been taken to gain insights of the viral structure, the clinical manifestations, and the immune responses caused by SARS‐CoV‐2. These movements can promote rapid development of therapeutic drugs, antibodies, and vaccines for this novel virus.

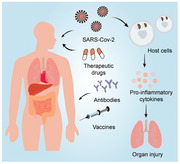

## 1 Introduction

Coronaviruses (CoVs) pose a significant threat to regional and global public health. Up to now, several CoVs have caused mild or severe diseases in humans and in wild animals. Human CoV infections impair the hepatic, digestive, respiratory, and central nervous systems.^[^ ^1^ ^]^ The severe acute respiratory syndrome (SARS) outbreak that occurred in China between 2002 and 2003 led to over 8000 human infections and 774 fatalities in 37 countries. Additionally, the Middle East respiratory syndrome (MERS) plagued the Middle East in 2012 and led to 2499 laboratory‐confirmed cases of infection and 861 deaths. Now, the novel CoV, severe acute respiratory syndrome coronavirus 2 (SARS‐CoV‐2), is responsible for the current pandemic and is infecting people globally, especially the elderly and those living with underlying comorbidities (e.g., diabetes and cardiovascular diseases).^[^ ^2^, ^3^, ^4^ ^]^ The ongoing SARS‐CoV‐2 pandemic has spread rapidly and has affected far more countries than SARS ever did.

Following the CoV disease 2019 (COVID‐19) outbreak, many practical and swift measures were taken to study this novel virus with the aim of preventing further spread.^[^ ^5^ ^]^ Genomic sequence analysis has shown that SARS‐CoV‐2 is very similar to several bat CoVs. Extensive research on the SARS‐CoV‐2 spike (S) protein, which recognizes the host receptor, has revealed the 3D structure of the protein and the unique furin recognition site within it. Additionally, accumulating clinical and experimental data has also revealed some common clinical manifestations and the host immune responses of people infected with SARS‐CoV‐2. However, the mechanism by which SARS‐CoV‐2 evades host recognition and suppresses host immunity remains unclear. Thus, in order to provide guidance for further study, we have reviewed the immune evasion strategies of SARS‐CoV and MERS‐CoV. Moreover, we summarize several potential treatment strategies for SARS‐CoV‐2 infection. We hope our review will offer clues for exploring the mechanisms used by SARS‐CoV‐2 to evade the human immune system, as well as aid in the diagnosis and treatment of SARS‐CoV‐2 infection.

## 2 The Genomic Structure of SARS‐CoV‐2

CoVs belong to the subfamily *Coronavirinae* in the family of *Coronaviridae* within the order *Nidovirales*, and are classified into four genera: *α*‐, *β*‐, *γ*‐, and *δ*‐CoVs. The *betacoronaviruses* can further be divided into four lineages (A–D) with SARS‐CoV and SARS‐CoV‐2 belonging to *betacoronavirus* lineage B. Several SARS‐CoV‐2 genomes have been identified and studies have shown that they have subtle differences in their genomes. A recent report based on 103 sequenced SARS‐CoV‐2 genomes suggested that these viruses could be divided into L‐ and S‐type based on the two linked single nucleotide polymorphisms (SNPs) at base pair 8782 and 28 144, with the former (≈70%) being more prevalent than the latter (≈30%). Interestingly, the S‐type genome was found to be the ancestral version.^[^ ^6^ ^]^ A comparison of the three determined SARS‐CoV‐2 genomes has shown that the virus contains 14 open reading frames (ORFs) which encode 27 viral proteins.^[^ ^7^ ^]^ ORF1a and ORF1b, which take up approximately two‐thirds of the whole genome, encode two polypeptides, pp1a and pp1ab. These two polypeptides are subsequently processed by a chymotrypsin‐like cysteine protease (3CL^pro^, also known as the main protease) and a papain‐like protease (PL^pro^), thereby producing multiple nonstructural proteins (nsp; nsp1–nsp10 and nsp12–nsp16^[^ ^7^, ^8^ ^]^). Other ORFs within the remaining third of the genome length encode four significant structural proteins: membrane (M), envelope (E), nucleocapsid (N), and S proteins, as well as eight accessory proteins (3a, 3b, p6, 7a, 7b, 8b, 9b, and orf14).^[^ ^7^ ^]^ Interestingly, similar to SARS‐CoV, the ORF8 gene is situated between the M and N genes in SARS‐CoV‐2.^[^ ^9^ ^]^ Some data have shown that in some SARS‐CoV‐2 genomes, a 382‐nt sequence which covers almost all of the entire ORF8 sequence is deleted and the ORF8 transcription‐regulatory sequence is altogether removed, thereby promoting the downstream transcription of the N gene.^[^ ^10^ ^]^ The structures and functions of the SARS‐CoV‐2 ORFs require further investigation.

The SARS‐CoV‐2 S protein is essential for successful invasion of the human body. Accordingly, many researchers have decided to study the structure of the trimeric S protein, which contains two subunits, S1 and S2, with the former binding to the host receptor while the latter mediates membrane fusion. The receptor‐binding domain (RBD) in the S1 subunit contains a core structure and a receptor‐binding motif which binds to the outer surface of the claw‐like structure of the angiotensin converting enzyme II (ACE2).^[^ ^11^ ^]^ As previously described, RBDs within lineage B of the CoVs could phylogenetically cluster into three clades. A recent study has shown that the SARS‐CoV‐2 RBD contains the majority of the human ACE2 contact points which are uncovered in clade 1, and some amino acid variations that are unique to clades 2 and 3, thereby suggesting that SARS‐CoV‐2 may have originated from recombination between clade 1 and the other clades.^[^ ^11^ ^]^ Furthermore, there are some data showing that the RBDs from clade 1 contain all 14 residues that interact with the human ACE2, and that most of these residues are absent from clades 2 and 3. Another study demonstrated that among the 14 contact points, 9 are completely conserved and 4 are partially conserved between SARS‐CoV‐2 and SARS‐CoV, further supporting the hypothesis that SARS‐CoV‐2 uses ACE2 as its receptor.^[^ ^12^ ^]^ The most surprising detail is linked with an insertion in the S1/S2 protease cleavage site that causes an “RRAR” furin recognition site in SARS‐CoV‐2, instead of the single arginine in SARS‐CoV, suggesting that SARS‐CoV‐2 may be more infective.^[^ ^13^ ^]^

**Figure 1:**
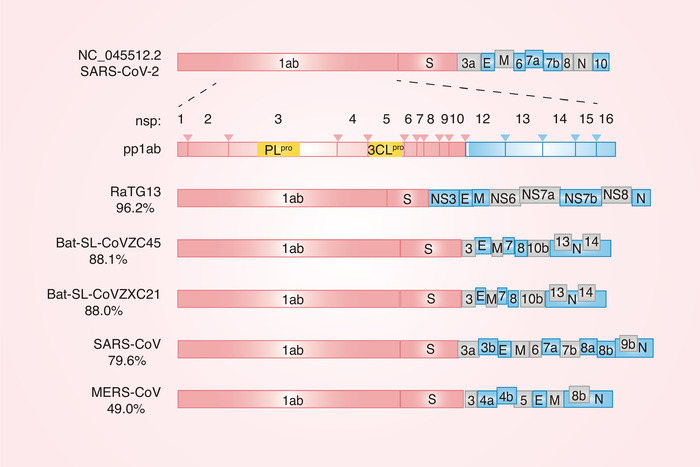
The structure of SARS‐CoV‐2. The genomic structure of SARS‐CoV‐2, Bat‐CoV RaTG13, Bat‐SL‐CoVZC45, Bat‐SL‐CoVZXC21, SARS‐CoV, and MERS‐CoV. The percentage below the name of CoVs refers to the similarity between that virus and SARS‐CoV‐2. ORF1a and ORF1b encode two polypeptides, pp1a and pp1ab. These two polypeptides are subsequently processed by PL^pro^ and 3CL^pro^, thereby producing multiple nonstructural proteins (nsp; nsp1–nsp10 and nsp12–nsp16). Nsp3 and nsp5 encode PL^pro^ and 3CL^pro^ activities, respectively.

SARS‐CoV‐2 is thought to have originated in bats as it shows 96.2% sequence identity to the genome of bat CoV RaTG13.^[^ ^14^ ^]^ Additionally, pangolin‐CoV is the second closest to SARS‐CoV‐2, with 91.02% identity at the whole‐genome level.^[^ ^15^, ^16^ ^]^ Interestingly, pangolin‐CoV and SARS‐CoV‐2 share five conserved critical RBD residues, which are involved in ACE2 binding, while RaTG13 only shares one amino acid with SARS‐CoV‐2 in this critical RBD, thereby suggesting that pangolin‐CoV could be pathogenically similar to SARS‐CoV‐2.^[^ ^16^, ^17^ ^]^ Other studies have shown that SARS‐CoV‐2 is also closely related to two bat‐derived CoV strains, bat‐SL‐CoVZC45 and bat‐SL‐CoVZXC21, which both share ≈88% sequence identity to SARS‐CoV‐2.^[^ ^18^ ^]^ Furthermore, the SARS‐CoV‐2 strains were less genetically similar to SARS‐CoV (about 79%) and MERS‐CoV (about 50%) at the whole‐genome level^[^ ^14^, ^18^ ^]^ (**Figure** **1**). A study of the amino acid substitutions in different proteins between SARS‐CoV‐2 and SARS‐CoV or SARS‐like bat CoVs revealed 380 amino acid substitutions: 61 and 102 amino acid substitutions in nsp2 and nsp3, respectively, and 27 amino acid substitutions in the S protein.^[^ ^7^ ^]^ However, whether these amino acid substitutions affect the pathogenicity of SARS‐CoV‐2 remains unclear.

## 3 Infection and RNA Synthesis of SARS‐CoV‐2

SARS‐CoV‐2 has a characteristic crown‐like appearance. The phospholipid bilayer on the external surface of the virus is embedded with densely glycosylated, homotrimeric class I fusion S proteins, all of which envelope the virion. The S proteins are also co‐localized with M (type III transmembrane glycoproteins) and E proteins. Inside is a nucleocapsid comprised of genomic RNA and phosphorylated N proteins. Structural proteins play critical roles in the viral life circle. For example, the M, E, and N proteins of SARS‐CoV are required for viral assembly, trafficking, and release and, importantly, the interactions between these structural proteins are key during the aforementioned processes.^[^ ^19^ ^]^

Much like SARS‐CoV, SARS‐CoV‐2 utilizes the S protein to invade host cells. The S protein is in a prefusion conformation that undergoes structural rearrangements intended for subsequent membrane fusion. First, RBDs in the S1 subunit undergo hinge‐like conformational changes that allow one of the three RBDs to assume the “up” conformation, thereby permitting easy access to the receptor, while the other RBDs adopt the “down” conformation making it difficult to make contact with the receptor.^[^ ^13^, ^20^ ^]^ This SARS‐CoV‐2 RBD movement is similar to what has been observed in SARS‐CoV with one of the notable differences being that the SARS‐CoV RBDs which are in the “down” conformation are near the N‐terminal domain (NTD), while in SARS‐CoV‐2 these are close to the center of the homotrimer.^[^ ^13^ ^]^ Second, the S1 subunit binds to a host cell receptor resulting in prefusion trimer instability and, presumably, strong infectivity. Third, the S1 subunit sheds. Subsequently, the heptad repeat 1 (HR1) and 2 (HR2) domains in the S2 subunit interact with each other to form a six‐helical bundle (6‐HB) fusion core, thereby triggering membrane fusion.^[^ ^21^ ^]^ Moreover, previous studies have shown that SARS‐CoV‐2 has an insertion in the S1/S2 protease cleavage site and that the virus utilizes host cell protease TMPRSS2 and cathepsin L for S protein priming, which may prepare the protein for membrane fusion via extensive irreversible conformational changes.^[^ ^22^, ^23^ ^]^

ACE2 acts as the cell receptor for SARS‐CoV.^[^ ^24^ ^]^ Recent studies have shown that SARS‐CoV‐2 can also bind to ACE2 from diverse animal hosts, except mice and rats, but does not bind to other CoV receptors including aminopeptidase N and dipeptidyl peptidase 4.^[^ ^12^, ^14^ ^]^ A recent study revealed that CD147, a transmembrane glycoprotein belonging to the immunoglobulin (Ig) superfamily, might also bind to S proteins to aid SARS‐CoV‐2 invasion of host cells.^[^ ^25^ ^]^ The SARS‐CoV‐2 S ectodomain has been shown to bind to ACE2 (*K*_D_ = 14.7 × 10^−9^ m) with ≈10–20‐fold higher affinity than the SARS‐CoV S ectodomain (*K*_D_ = 325 × 10^−9^ m).^[^ ^13^ ^]^ However, other studies have revealed that the binding affinities between ACE2 and SARS‐CoV‐2 (≈10 × 10^−9^ to 60 × 10^−9^ m) are comparable to those of ACE2 and SARS‐CoV RBDs.^[^ ^26^, ^27^ ^]^ Full‐length ACE2 consists of an N‐terminal peptidase domain (PD) which binds to the RBD and a C‐terminal collectrin‐like domain (CLD) which is composed of the Neck domain and a single transmembrane helix. The structure of the full‐length ACE2 receptor stabilized by a neutral amino acid transporter B^0^AT1 was recently reported and showed that ACE2 served as a homodimer to accommodate two S protein trimers. The conformational changes induced in the ACE2 (i.e., open and closed conformations) are achieved through rotation of the PD domains, while the interface between the Neck domains remains unchanged.^[^ ^28^ ^]^ ACE2 is highly expressed in lung alveolar type II(AT2) cells, esophagus epithelial cells, and absorptive enterocytes from the ileum and colon.^[^ ^29^, ^30^ ^]^ Additionally, ACE2 can also be found in renal tubular cells, Leydig cells, as well as cells in seminiferous ducts in the testes.^[^ ^31^ ^]^ Furthermore, smokers show upregulated *ACE2* gene expression compared to nonsmokers, which may predispose smokers to SARS‐CoV‐2.^[^ ^2^, ^32^ ^]^ Type I interferons (IFNs) are considered as the most essential antiviral cytokines and can suppress CoVs infection if timely present and properly localized. Thus, many CoVs evolved mechanisms to inhibit the IFN‐I expression. However, it is noticed that infection of SARS‐CoV‐2 accompanied by IFNs, upregulates ACE2 protein expression, thereby facilitating the spread of the virus.^[^ ^33^ ^]^ Besides, delayed IFN‐I may contribute to the severe disease.^[^ ^34^ ^]^ This seemingly contradictory phenomenon is worthy of further exploration.

**Figure 2:**
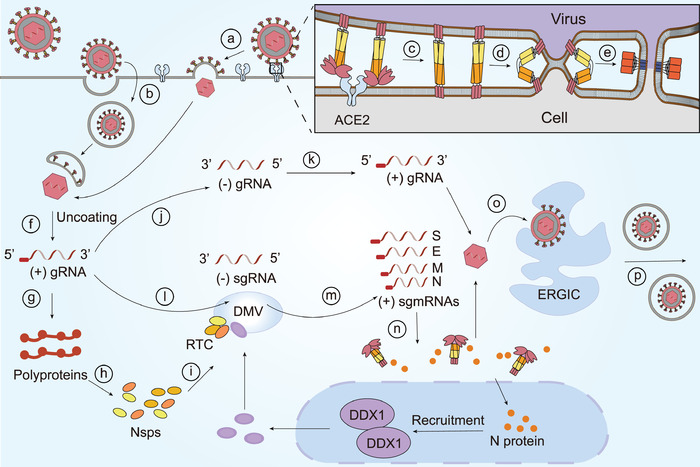
The fusion steps and RNA synthesis process of CoVs. CoVs can fuse either directly to the a) plasma membrane or b) after endocytosis. The membrane fusion process is complex: c) the S1 subunit that has experienced conformation rearrangements bounds to a host cell receptor and later sheds; d) S2 triggers two bilayers into one; e) subsequently, HR1 and HR2 domains in S2 subunit form a 6‐HB fusion core, causing membranes fusion. After entry, f) genomic RNA (gRNA) is released from the viral coat and g) serves as the template for translation of polyproteins which h) encode nsps to form the i) RTC in DMVs where CoVs RNA synthesis is performed. Some CoVs N protein transited to the nucleus could recruit the helicase DDX1 which functioned in the RTC to facilitate synthesis of sgmRNA. j) CoVs utilize a whole‐length complementary negative‐strand RNA to k) replicate. However, CoVs produce sgmRNAs in a discontinuous manner. l) Genomic RNA serves as template to form negative‐strand subgenomic RNAs (sgRNA), m) which are subsequently utilized for the synthesis of multiple copies of sgmRNAs that will later n) encode viral structural proteins and accessory proteins. Eventually, viral gRNA and significant proteins assemble into viral particles in o) the ER–Golgi intermediate complex (ERGIC) which are then p) secreted to the outside of cells via the secretory pathway. Upper‐right: S1 colored pink, HR1 colored orange, HR2 colored yellow, and 6‐HB colored red.

SARS‐CoV‐2 enters 293/human ACE2 cells mainly through endocytosis in which phosphatidylinositol 3‐phosphate 5‐kinase (PIKfyve) and two pore channel subtype 2 (TPC2) are critical.^[^ ^23^ ^]^ Normally, when gaining entry into the host cells, CoVs elaborately modify intracellular membranes to form double‐membrane vesicles (DMVs).^[^ ^35^, ^36^ ^]^ CoV RNA synthesis is performed by a replication–transcription complex (RTC) that includes viral and cell proteins. Some CoV N proteins are transported to the nucleus to recruit the helicase DDX1 that functions in the RTC to facilitate synthesis of subgenomic mRNAs (sgmRNA).^[^ ^35^, ^37^ ^]^ The replication processes of other CoVs have been reviewed elsewhere,^[^ ^35^, ^38^ ^]^ and SARS‐CoV‐2 replication may be similar to other CoVs (**Figure** **2** and **Table** **1**).

**Table 1:** The processes during SARS‐CoV‐2 infection

Stage	Process
Membrane fusion	RBDs in the S1 subunit experience the conformational changes
	The S1 subunit binds to a host cell receptor, resulting in prefusion trimer instability
	The S1 subunit sheds
	The S2 subunit triggers two bilayers into one
	HR1 and HR2 domains in the S2 subunit interact with each other to form 6‐HB fusion core
Viral replication and translation	Genomic RNA is released from the viral coat
	Genomic RNA serves as the template for translation of polyproteins
	Polyproteins encode nsps to form the RTC in DMVs
	Genomic RNA replicates continuously by utilizing a negative‐strand RNA as the template
	Genomic RNA serves as template to form negative‐strand sgRNAs
	SgRNAs are utilized for the synthesis of multiple copies of sgmRNAs
	SgmRNAs encode viral structural proteins and accessory proteins
Assembly and release	Genomic RNA and significant proteins assemble into the mature viral particles
	Viral particles are secreted to the outside of cells

## 4 Clinical Symptoms

SARS‐CoV and MERS‐CoV infect the lower airway to cause severe respiratory syndrome in humans.^[^ ^39^ ^]^ Previous reports have suggested that the case fatality rates (CFRs) of MERS‐CoV, SARS‐CoV, and SARS‐CoV‐2 were 35%, 9.6%, and 2.3%, respectively. However, the CFR of SARS‐CoV‐2 increases significantly in those with preexisting comorbidities—10.5% for cardiovascular disease, 7.3% for diabetes, 6.3% for chronic respiratory disease, 6.0% for hypertension, and 5.6% for cancer.^[^ ^40^ ^]^ The literature shows that the most common symptoms at the onset of SARS‐CoV‐2 infection are fever, unproductive cough, and myalgia or fatigue; less common symptoms include headache, sputum production, hemoptysis, and diarrhea.^[^ ^40^, ^41^, ^42^, ^43^ ^]^ With deterioration of clinical status, patients infected with SARS‐CoV‐2, especially those in the intensive care unit (ICU), who require high‐flow oxygen therapy are likely to suffer from hypoxemia and require invasive ventilation or noninvasive ventilation as a treatment.^[^ ^41^, ^42^, ^43^ ^]^

Compared to healthy people, serological testing in SARS‐CoV‐2‐infected patients shows dysregulation of blood urea nitrogen, serum creatinine, and creatine kinase. Patients also show high levels of aspartate aminotransferase and alanine aminotransferase, indicating some level of liver function abnormality.^[^ ^41^, ^42^, ^43^ ^]^ Notably, a comparison of some indexes between ICU and non‐ICU patients shows that the concentrations of prothrombin D‐dimer and aspartate aminotransferase are much higher in ICU patients than in non‐ICU patients, suggestive of a degree of the viral infection.^[^ ^41^, ^43^ ^]^ Several studies revealed that SARS‐CoV‐2 infection may lead to lymphocytopenia.^[^ ^41^, ^44^, ^45^ ^]^ Of note, eosinophil counts were reduced in some patients and had positive correlation with lymphocyte numbers.^[^ ^45^ ^]^

### 4.1 Lung Pathology of SARS‐CoV‐2 Infection

Most chest computed tomography (CT) images showed notable abnormalities with airspace shadowing that were detected in most patients infected with SARS‐CoV‐2. Thus, CT is a vital tool for SARS‐CoV‐2 diagnosis. The representative chest CT findings contain bilateral and peripheral pneumonia, ground‐glass opacity and patchy consolidation, with the right lower lobe being more susceptible to the infection.^[^ ^3^, ^42^, ^45^, ^46^, ^47^, ^48^ ^]^ Other changes include multiple mottling, pneumothorax, pleural effusions, lymphadenopathy, bronchiolectasis, nodules, and cystic changes, which rarely occur.^[^ ^42^, ^46^, ^47^, ^49^ ^]^ Ultimately, the patients’ health condition is synchronously reflected on CT images. Studies have shown that ICU patients suffering from severe pneumonia typically show multiple lobar and subsegmental areas of consolidation in both lungs, while non‐ICU patients showed bilateral ground‐glass opacity and subsegmental areas of consolidation.^[^ ^41^, ^50^ ^]^ Some SARS‐CoV‐2‐infected individuals may deteriorate rapidly and develop acute respiratory distress syndrome (ARDS), usually accompanied by diffuse alveolar damage (DAD). Recent reports of autopsies aid us in better understanding the pathology of severe patients. Autopsy of a 77‐year‐old obese man showed DAD and chronic inflammation and edema in the bronchial mucosa.^[^ ^51^ ^]^ A case report from China also described DAD with cellular fibromyxoid exudates and noticed the interstitial mononuclear inflammatory infiltrates in postmortem biopsies.^[^ ^52^ ^]^ Another postmortem examinations exhibited advanced DAD in all biopsies, and superimposed bacterial pneumonia in one.^[^ ^53^ ^]^ However, the knowledge concerning the pathogenesis of SARS‐CoV‐2 infection is still limited, which requires more investigation.

## 5 Host–Pathogen Interactions

### 5.1 Immune Responses During SARS‐CoV‐2 Infection

During SARS‐CoV infection, a delayed but overactive production of cytokines and chemokines induces dysregulated innate immune responses, resulting in exacerbation of symptoms in patients.^[^ ^54^ ^]^ Like SARS‐CoV, SARS‐CoV‐2 infection leads to changes in cytokines and chemokines that might correlate with the inflammatory and immune responses of the infected patients. A study by Huang et al. in 2020 reported increased expression of interleukin‐1*β* (IL1*β*), IL1RA, IL7, IL8, IL9, IL10, IFN*γ*, interferon gamma‐induced protein 10 (IP10), monocyte chemoattractant protein‐1 (MCP1), macrophage inflammatory protein (MIP), and tumor necrosis factor‐alpha (TNF*α*) in SARS‐CoV‐2 patients after onset of illness. The expression of IL2, IL7, IL10, IP10, MCP1, MIP1A, and TNF*α* were much higher in more severe patients.^[^ ^41^ ^]^ Hence, cytokine counts may be associated with the disease severity. Intriguingly, unlike SARS‐CoV, SARS‐CoV‐2 infection also initiates increased secretion of anti‐inflammatory cytokine IL10 that may explain the divergence in the immunological lung injury caused by the two viruses.^[^ ^55^, ^56^ ^]^ A recent study showed remarkably elevated levels of inflammatory monocyte, plasmacytoid dendritic cells, and plasmablasts, and showed increased expression of genes which are critical for antiviral activity (i.e., *DDX58*, *IFIH1*, *DHX58*, *TLR7*, *TLR8*, *MAVS*, *IRF3*, and *IRF7*) in the peripheral blood mononuclear cells (PBMCs) of ICU patients’ compared to pre‐ICU and post‐ICU samples.^[^ ^57^ ^]^ Additionally, IFN‐I, especially IFN‐*α*, was hypothesized to be one of the major cytokines mediating host immune defense in severe SARS‐CoV‐2 patients.^[^ ^57^ ^]^ However, additional experiments showed that SARS‐CoV‐2 elicited a muted response that lacked robust induction of a subset of cytokines including IFN‐I and IFN‐III. The study also noted the upregulation of two unique cytokines, EDN1 and TNFSF15, during SARS‐CoV‐2 infection compared to the responses to other respiratory viruses.^[^ ^58^ ^]^ Notably, SARS‐CoV‐2 can cause the cytokine storms, while several viral proteins may be able to suppress the nuclear factor kappa‐light‐chain‐enhancer of activated B cells (NF‐kB) pathway. More efforts are expected to be taken in studying the mechanism underlying such phenomenon.

Lymphopenia is another symptom in SARS‐CoV‐2 infected patients, especially those with severe infection, suggesting an impaired adaptive immune response.^[^ ^41^, ^44^, ^59^ ^]^ In fact, SARS‐CoV‐2‐mediated impairment of secondary lymphoid organs such as the spleen and lymph nodes was partially responsible for lymphopenia. Some have suggested that this impairment occurs by infection of tissue‐resident CD169^+^ macrophages, which have ACE2 and lymphocyte apoptosis through enhancing Fas expression.^[^ ^60^ ^]^ Moreover, the number of CD4^+^ T cells is significantly reduced in mild and severe patients compared to healthy people, while CD8^+^ T cell numbers are markedly reduced in severe patients.^[^ ^61^, ^62^ ^]^ No significant changes in numbers have been observed in B cells and NK cells in SARS‐CoV‐2 infected patients compared to healthy individuals.^[^ ^61^, ^62^ ^]^ Interestingly, the numbers of both T and B cells are known to decline with age, which may explain why the elderly people are more susceptible to SARS‐CoV‐2.^[^ ^59^ ^]^ Despite lymphocyte depletion, CD4^+^ T lymphocytes are swiftly activated to become pathogenic T helper (Th) 1 cells with increased expression of granulocyte‐macrophage colony stimulating factor (GM‐CSF) and IL‐6. Additionally, there seems to be a high degree of Tim3^+^ PD‐1^+^ co‐expression in CD4^+^ and CD8^+^ T cells, indicative of T‐cell exhaustion in infected SARS‐CoV‐2 patients.^[^ ^62^ ^]^ NKG2A expression was markedly upregulated in NK and CD8^+^ T cells, also indicative of NK and CD8^+^ T cell functional exhaustion.^[^ ^63^ ^]^ In addition, although the numbers of monocytes decrease significantly in SARS‐CoV‐2‐infected patients compared to healthy individuals, the cytokine microenvironment activates CD14^+^CD16^+^ inflammatory monocytes leading to IL‐6 secretion and exacerbation of the cytokine storm.^[^ ^62^ ^]^ A recent report on a mild patient revealed that the patient exhibited increased expression of antibody‐secreting cells, follicular helper T cells (T_FH_ cells), CD38^+^HLA‐DR^+^ CD4^+^ T cells, CD8^+^ T cells, and IgM and IgG antibodies which bind to SARS‐CoV‐2 in the bloodstream before symptomatic recovery.^[^ ^64^ ^]^ However, some studies have shown that severe patients infected with SARS‐CoV‐2 frequently exhibited a more robust IgG response and higher antibody titers, which correlated with worse clinical outcome, suggestive of possible antibody‐dependent enhancement of SARS‐CoV‐2 infection.^[^ ^65^, ^66^ ^]^ Overall, further investigations are required to characterize immune responses to SARS‐CoV‐2 and the underlying mechanisms are essential to aid our understanding of COVID‐19.

### 5.2 Potential Immune Evasion Strategies

**Figure 3:**
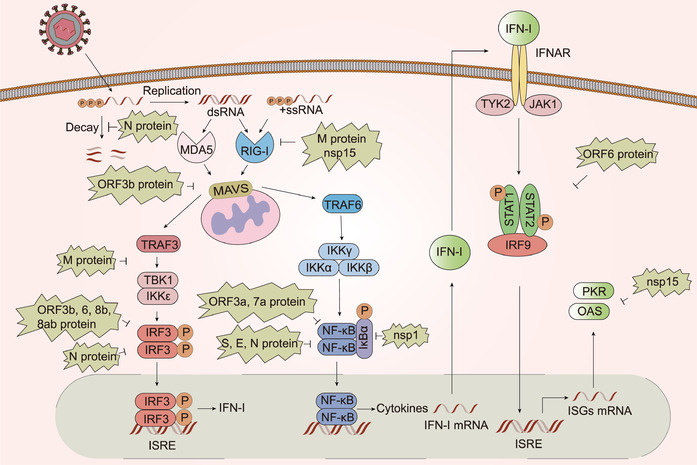
Type I interferon pathway during SARS‐CoV infection and modulatory mechanisms, PL^pro^ excluded. MDA5 and RIG‐I can both recognize SARS‐CoV dsRNA and RIG‐I is also able to recognize uncapped 5′‐triphosphate RNA. M protein can avoid IFN production mediated by RIG‐I and disrupt the interaction of TRAF3 with TANK, TBK1, and IKKɛ. N protein may serve as an NMD inhibitor and is able to interfere IRF3 signaling pathway. Besides, nsp15 possesses EndoU activity that can make virus avoid recognized by MDA5, PKR, and OAS‐RNase L pathway. Additionally, S, E, and N protein, as well as nsp1 can antagonize NF‐ kB pathway. Besides, ORF3b, 6, 8b, and 8ab can inhibit mitochondrial antiviral signaling protein (MAVS)‐mediated IRF3 pathway, while ORF3a and ORF 7a protein are able to suppress NF‐kB pathway. ORF6 protein can also inhibit STAT1 nuclear import in response to IFN signaling.

To better adapt to a hostile environment, viruses usually develop a myriad of unique strategies to escape the host immune response and to suppress the host immune system (**Figure** **3**). The virulence of human infections is associated with immune evasion strategies. Up to now, there have been few studies regarding the mechanisms by which SARS‐CoV‐2 evades the immune system. Nonetheless, the insights we have gained into the immune evasion mechanisms of SARS‐CoV and MERS‐CoV may serve as guides in the study of SARS‐CoV‐2 immune suppression.

The human immune system is comprised of two interrelated parts, innate immune responses and adaptive immune responses. As the first line against virus infection, the innate immune response employs pattern recognition receptors to detect common pathogenic molecular features.^[^ ^38^, ^67^ ^]^ Double‐stranded RNA (dsRNA), a replication intermediate of positive‐stranded RNA viruses, is an important pathogen‐associated molecular pattern that is recognized by cytoplasmic retinoic acid‐inducible gene I (RIG‐I) and melanoma differentiation‐associated protein 5 (MDA5). RIG‐I also recognizes uncapped 5′‐triphosphate RNA.^[^ ^68^ ^]^ Host cell responses include IFN‐I expression, the inhibition of host cell translation by activating protein kinase R (PKR), and the degradation of viral and host cell‐derived RNA by activating the oligoadenylate synthetase (OAS)/RNase L pathway. PKR is a kinase that can be directly activated by dsRNA to phosphorylate the eukaryotic initiation factor 2*α* (eIF2*α*), resulting in translation inhibition of cellular and viral mRNAs, while the OAS‐RNase L pathway plays a critical role in RNA degradation. The innate immune responses are essential for subsequent activation of the adaptive immune response. The adaptive immune system can be briefly classified into two branches: the humoral immune response arm (i.e., production of antibodies by B cells) and the cellular immune response arm (i.e., activities undertaken by CD4^+^ and CD8^+^ T cells).

Nsp1, the 5′‐terminal subunit of the replicate polyprotein, has been shown to bind to cellular factors of the translation machinery, thereby preventing translation of host mRNAs. For instance, SARS‐CoV nsp1 interacts with the 40S ribosomal subunit and the nsp1‐40S ribosome complex subsequently modifies the 5′ regions of capped mRNA templates, thereby rendering them translationally incompetent.^[^ ^69^ ^]^ Nevertheless, the specific interaction of nsp1 with the 5′ untranslated regions (UTRs) of SARS‐CoV RNAs confers resistance to the nsp1‐mediated translational shutoff and prompts viral RNA replication, since the structure of stem‐loop 1 (SL1) in the 5′UTR of SARS‐CoV is involved in the discrimination between viral (both genomic and subgenomic) RNAs and host mRNAs.^[^ ^70^ ^]^ Additionally, SARS‐CoV nsp1 has been shown to block signal transducer and activator of transcription 1 (STAT1) phosphorylation and IFN regulatory factor (IRF3) dimerization.^[^ ^71^, ^72^ ^]^

**Figure 4:**
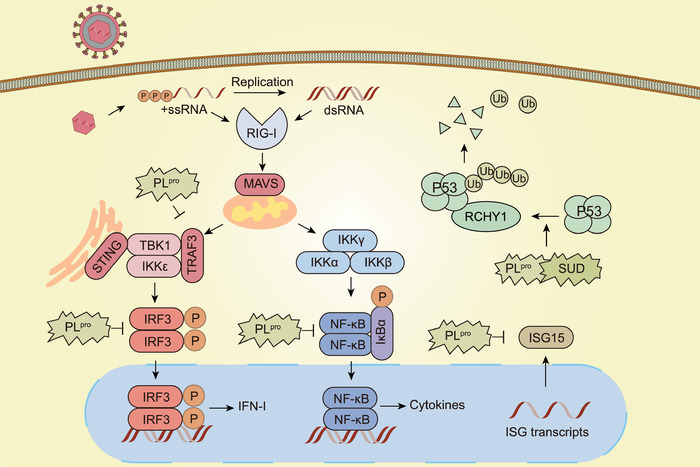
The functions of PL^pro^ in modulating host immune system and inhibiting IFN‐I signaling during SARS‐CoV infection. PL^pro^ could inhibit the ubiquitination of RIG‐I, TRAF3, STING, TBK1, and IRF3, and disrupted the assembly of STING‐TRAF3‐TBK1 complex. Besides, it could suppress IRF3 phosphorylation, dimerization, and nuclear translocation, as well as suppress activation of the NF‐kB. PL^pro^ also causes ISG15 deconjugation. Additionally, SUD and PL^pro^ of SARS‐CoV could interact with and stabilize RCHY1, triggering the degradation of p53 that functions as a host defense molecule during viral infection.

PL^pro^ is essential for CoV replication, as well as processing the viral replicase polyproteins, and it functions as a potent IFN antagonist (**Figure** **4**). The IFN‐induced ubiquitin‐like molecule, ISG15, functions as a protein modifier in transcription and pre‐mRNA splicing during IFN responses.^[^ ^73^ ^]^ PL^pro^ is expressed in SARS‐CoV and shares structural similarities with the herpesvirus‐associated ubiquitin‐specific protease, a deubiquitinating enzyme. Thus, CoV PL^pro^ may exhibit deubiquitinating activities leading to ISG15 deconjugation.^[^ ^74^, ^75^ ^]^ Furthermore, the SARS‐unique domain (SUD) and PL^pro^ from SARS‐CoV nsp3 as well as PL^pro^ from MERS‐CoV and HCoV‐NL63 have been shown to interact with and stabilize E3 ligase ring‐finger and CHY zinc‐finger domain‐containing 1 (RCHY1) via a yet, uncharacterized mechanism. This interaction triggers the degradation of endogenous p53, which serves as a host defense molecule during viral infection.^[^ ^76^ ^]^ Moreover, PL^pro^ interacts with IRF3 to inhibit its phosphorylation, dimerization, and nuclear translocation in a protease‐independent manner, thereby counteracting the production of IFN‐I.^[^ ^77^ ^]^ It was further uncovered that SARS‐CoV PL^pro^ inhibited the ubiquitination of RIG‐I, TNF receptor‐associated factor 3 (TRAF3), stimulator of interferon genes protein (STING), TANK binding kinase 1(TBK1), and IRF3 and disrupted the assembly of STING‐TRAF3‐TBK1 complex,^[^ ^78^ ^]^ which also suggested that it might broadly target the type I interferon pathway to serve as a powerful interferon antagonist. In addition to IRF3 signaling, PL^pro^ also disrupts NF‐kB signaling.^[^ ^79^ ^]^

The RNA replication of some CoVs generally occurs in DMVs. Several lines of evidence have shown that for MERS‐CoV and SARS‐CoV, co‐expression of nsp3 and nsp4 is required and ample for DMV formation. As viral RNAs are localized in DMVs, they remain unreachable by the innate immune sensors of the cytosol.^[^ ^80^ ^]^ Although it was assumed by many researchers that DMVs shield CoVs from innate immune recognition, few reports have investigated the phenomenon in any depth.

The nonstructural CoV protein, nsp15, retains highly conserved endonuclease (EndoU) activity and several studies have suggested that CoV EndoU activity is crucial for preventing early host immune responses. One study showed that replication of EndoU‐deficient mutants of HCoV‐229E was severely restricted and that elevated IFN‐I expression was observed in human macrophages. The underlying mechanism behind this observation may be caused by CoV EndoU‐mediated inhibition of dsRNA sensors, such as MDA5, PKR, and the OAS‐RNase L pathway.^[^ ^81^ ^]^ Thus, CoVs EndoU inhibitors may be promising antiviral agents as they could potentially restore efficient sensing of viral RNA, thereby activating antiviral effector pathways (i.e., IFN‐I, PKR, and OAS/RNase L).

A 5’‐cap structure is essential for CoV survival in human beings. CoVs have evolved mechanisms to modify their cap structure that play a critical role in escaping host immune recognition. SARS‐CoV nsp16 has been shown to methylate the RNA cap at ribose 2′‐O and utilize nsp10 as a stimulatory factor to execute its methyltransferase activity.^[^ ^82^, ^83^ ^]^ Another study demonstrated that additional 2’‐O‐methylation of 5’‐cap structures helps CoVs to avoid recognition by MDA5 and subsequent stimulation of IFN‐I.^[^ ^84^ ^]^ Moreover, 2′‐O‐methylation of viral mRNA might also assist evasion of IFN‐mediated restriction of viral replication.^[^ ^84^ ^]^

In addition to nonstructural CoV proteins, structural proteins, although less conserved, are detrimental to the host innate immune system. Coronaviral mRNAs are targeted by the cellular nonsense‐mediated decay (NMD) pathway, an eukaryotic RNA surveillance pathway that detects aberrant mRNA structures such as premature stop codons, multiple ORFs with internal stop codons, and a long 3′ UTR, and thus causes in viral mRNA degradation.^[^ ^85^, ^86^ ^]^ To overcome this problem, CoVs have evolved multifunctional proteins to escape NMD recognition. For example, some CoV N proteins were shown to serve as NMD inhibitors.^[^ ^86^ ^]^ Therefore, we should investigate whether SARS‐CoV‐2 has employed a similar mechanism to perturb the NMD pathway. Additionally, the SARS‐CoV N protein has been shown to impede IFN synthesis by inhibiting IRF3 and NF‐*κ*B.^[^ ^87^ ^]^ The M protein of some CoVs has also been proven to play a significant role in the evasion of the host immune system. The SARS‐CoV M protein has been shown to inhibit RIG‐I‐mediated, but not MDA5‐mediated, IFN production in infected HEK293 cells.^[^ ^88^ ^]^ In addition, the M protein also disrupts the interaction between TRAF3 and TANK, TBK1, and I*κ*B kinase ɛ (IKKɛ) through its first transmembrane domain (TM1), which is located at the N‐terminus, thereby inhibiting activation of IRF3/IRF7.^[^ ^89^, ^90^ ^]^ Similarly, the MERS‐CoV M protein was shown to inhibit IRF3 activity by disrupting the interaction between TRAF3 and TBK1.^[^ ^91^ ^]^ Moreover, the E and S proteins of SARS‐CoV have been reported to interfere NF‐*κ*B signaling.^[^ ^88^ ^]^ Whether the M, E, and S proteins of SARS‐CoV‐2 possess comparable functions remains unknown.

Accessory proteins have also been shown to contribute to host immune modulation. The ORF3b protein of SARS‐CoV has a unique shuttling behavior. Initially, ORF3b is located in the nucleus and is subsequently translocated to the outer mitochondrial membrane in a leptomycin B‐sensitive dependent nuclear export mechanism, thereby subverting RIG‐I‐mediated IFN induction and the mitochondrial antiviral signaling proteins.^[^ ^92^ ^]^ Although SARS‐CoV ORF6 does not prevent the phosphorylation of STAT1, it does hinder STAT1 from localization into the nucleus in response to IFN‐I, thereby blocking the expression of STAT1‐activated genes that establish an antiviral state.^[^ ^87^, ^93^ ^]^ Furthermore, SARS‐CoV ORF3b, ORF6, ORF8a, and ORF8ab have been identified as effective inhibitors of IRF‐3 phosphorylation, resulting in suppressed IFN‐*β* expression.^[^ ^38^, ^87^, ^88^ ^]^ Moreover, SARS‐CoV ORF3a and ORF7a are implicated in the inhibition of the NF‐*κ*B signaling pathway.^[^ ^88^ ^]^ Although SARS‐CoV‐2 may have different accessory proteins, it is likely that SARS‐CoV‐2 accessory proteins have similar roles in immune evasion, which merits further study.

In terms of adaptive immune evasion, MERS‐CoV causes T‐cells apoptosis by activating intrinsic and extrinsic apoptosis pathways.^[^ ^94^ ^]^ The SARS‐CoV E protein may induce T‐cell apoptosis via a pathway antagonistic to the mitochondrion‐dependent mechanism of the antiapoptotic protein Bcl‐xL.^[^ ^95^ ^]^ In addition, previous studies have shown that MERS‐CoV infection of macrophages or dendritic cells results in decreased antigen presentation through major histocompatibility complex (MHC) class I and MHC class II, thereby significantly affecting T‐cell activation.^[^ ^96^ ^]^ SARS‐CoV infection also actively suppressed the induction of adaptive immunity by impairing macrophage and dendritic cell functions.^[^ ^97^ ^]^ T‐cell depletion and the failure to present antigens may pose a real threat in delaying and weakening the onset of the adaptive immune responses against viral infection.

Taken together, the data discussed here suggest that CoVs have and are likely to evolve additional diverse immune evasion mechanisms to further improve infectivity in a hostile environment. Given that SARS‐CoV‐2 is phylogenetically related to CoVs of bat origin, the insights gained into other CoV immune evasion tactics can guide future SARS‐CoV‐2 studies as we need to learn more about this novel CoV.

## 6 Potential Therapeutic and Preventive Methods

### 6.1 Virus‐Based Treatment Approaches: Viral Enzyme‐Targeting Drugs

As CoVs can manipulate the host immune system, therefore, it is plausible that these viruses may destroy pre‐existing natural or vaccine inflicted immunity, thereby establishing an obstacle in the development of efficient CoV therapies. Following the initial SARS‐CoV‐2 outbreak in Wuhan, Central China, a number of rapid measures were adopted to develop the most effective and suitable drugs against SARS‐CoV‐2. Other actions were also taken to evaluate the potential value of predicted commercial antiviral drugs that may also inhibit SARS‐CoV‐2 infection (**Table** **2**). Under such difficult circumstances, testing whether the existing antiviral drugs are effective in treating COVID‐19 is a time‐saving and cost‐effective approach.

**Table 2:** Potential therapeutic and preventive approaches

Antiviral agents	Other treatable diseases	Drug targets	Potent mechanisms of inhibiting SARS‐CoV‐2	Ref.
***Virus‐based treatment approaches***				
*Viral enzymes‐targeting drugs*				
Lopinavir	HIV	3CLpro	Forms 2 hydrogen bonds with 3CLpro	^[^ ^98^, ^99^, ^100^ ^]^
Ritonavir	HIV	3CLpro	Forms 2 hydrogen bonds with 3CLpro	^[^ ^98^, ^99^, ^100^ ^]^
Colistin	Antibiotic	3CLpro	Forms 9 hydrogen bonds with 3CLpro	^[^ ^101^ ^]^
Valrubicin	Tumor	3CLpro	Forms 7 hydrogen bonds with 3CLpro	^[^ ^101^ ^]^
Lcatibant	Hereditary angioedema	3CLpro	Forms 6 hydrogen bonds with 3CLpro	^[^ ^101^ ^]^
Bepotastine	Rhinitis, uriticaria	3CLpro	Forms 5 hydrogen bonds with 3CLpro	^[^ ^101^ ^]^
Epirubicin	Tumor	3CLpro	Forms 4 hydrogen bonds with 3CLpro	^[^ ^101^ ^]^
Epoprostenol	Vasodilator	3CLpro	Forms 4 hydrogen bonds with 3CLpro	^[^ ^101^ ^]^
Vapreotide	Tumor	3CLpro	Forms 3 hydrogen bonds with 3CLpro	^[^ ^101^ ^]^
Aprepitant	Tumor	3CLpro	Forms 3 hydrogen bonds with 3CLpro	^[^ ^101^ ^]^
Caspofungin	Antifungal	3CLpro	Forms 3 hydrogen bonds with 3CLpro	^[^ ^101^ ^]^
Perphenazine	Psychotic	3CLpro	Forms 2 hydrogen bonds with 3CLpro	^[^ ^101^ ^]^
Prulifloxacin	Urinary tract infection	3CLpro	Exhibits three binding sites with 3CLpro	^[^ ^102^ ^]^
Tegobuvir	HCV	3CLpro	Binds to the joint groove of 3CLpro	^[^ ^102^ ^]^
Bictegravir	HIV	3CLpro	Binds to the joint groove and the active sites of 3CLpro	^[^ ^102^ ^]^
Nelfinaviras	HIV	3CLpro	Binds to the joint groove of 3CLpro	^[^ ^102^ ^]^
*α*‐ketoamide	/	3CLpro	Inhibits 3CLpro	^[^ ^103^ ^]^
11a and 11b	/	3CLpro	Binds to the substrate‐binding pocket of SARS‐CoV‐2 3CLpro	^[^ ^104^ ^]^
Atazanavir	HIV	RdRp, 3′‐to‐5′ exonuclease, RNA helicase, endoRNAse, 2′‐O‐ribose methyltransferase	Inhibits viral replication process at multiple level	^[^ ^105^ ^]^
Ganciclovir	HSV	RdRp, 3′‐to‐5′ exonuclease, RNA helicase	Inhibits viral replication process	^[^ ^105^ ^]^
Darunavir and Cobicistat	HIV	Helicase	Binds to helicase	^[^ ^105^ ^]^
Galidesivir	Broad‐spectrum viruses infection	RdRp	Terminates an RNA chain	^[^ ^106^ ^]^
Remdesivir	Ebola	RdRp	Terminates an RNA chain	^[^ ^106^, ^107^, ^108^, ^109^ ^]^
Favipiravir	Influenza	RdRp	Inhibits viral replication process	^[^ ^110^ ^]^
*Antispike protein therapies*				
47D11 antibody	SARS‐CoV infection	RBDs	Targets the conserved core structure of the S1 RBD of SARS‐CoV‐2	^[^ ^113^ ^]^
VHH‐72‐IgG Fc	SARS‐CoV S pseudotyped viruses	S protein	Disrupts RBD dynamics and receptor‐binding, and neutralizes SARS‐CoV‐2 S pseudoviruses	^[^ ^114^ ^]^
EK1C4	SARS‐CoV, MERS‐CoV, HCoV‐OC43, HCoV‐NL63, and HCoV‐229E	HR1 domain	Inhibits cell–cell fusion and pseudovirus infection	^[^ ^115^ ^]^
IPB02	SARS‐CoV pseudotyped viruses	A prehairpin intermediate	Binds to S2 subunit that is in a prehairpin intermediate	^[^ ^116^ ^]^
Recombinant ACE2 protein	SARS‐CoV infection	S protein	Inhibits virus entry to cells	^[^ ^118^ ^]^
*Other potent therapies*				
CRISPR/Cas13d system	Broad‐spectrum viruses infection	The viral genome	Inhibits viral replication process	^[^ ^120^ ^]^
CVL218	/	PARP1	Inhibits the viral multiplication and suppresses the CpG‐induced IL‐6 production	^[^ ^121^ ^]^
***Host‐based treatment approaches***				
Teriflunomide	Sclerosis	DHODH	Interferes pyrimidine synthesis and eliminates cytokine storm	^[^ ^122^ ^]^
Carolacton	/	MTHFD1	Inhibits purine synthesis	^[^ ^123^ ^]^
Chloroquine	Malarial	Endosomal acidifications	Increases endosomal pH	^[^ ^107^ ^]^
Hydrochloroquine	Malarial	Endosomal acidifications	Reduces the respiratory viral load and lessens the duration of SARS‐CoV‐2 carriage	^[^ ^126^, ^127^ ^]^
Tocilizumab	Giant cell arteritis, vasculitis	IL‐6 receptor	Curbs immunopathology driven by SARS‐CoV‐2	^[^ ^62^, ^128^ ^]^
Meplazumab	Asthma	CD147	Inhibits viral entry into host cells and binds with cyclophilin A	^[^ ^130^ ^]^
Baricitinib	Rheumatoid arthritis	AAK1, JAK, and GAK	Reduces inflammation and inhibits viral entry	^[^ ^131^, ^132^ ^]^
Mesenchymal stem cells	Graft versus‐host disease and systemic lupus erythematosus	Immune cells	Reduces cytokine storm	^[^ ^133^, ^134^ ^]^
Corticosteroids	SARS‐CoV infection	Immune cells	Inhibits the production of cytokines and viral replication	^[^ ^135^, ^136^ ^]^
Convalescent plasma	Influenza A, SARS‐CoV, and Ebola virus	/	Suppresses virus to enter into cells	^[^ ^137^, ^138^, ^139^ ^]^
Vaccines	Broad‐spectrum viruses infection	/	Induces antibodies against virus	^[^ ^141^, ^142^, ^143^, ^144^, ^145^ ^]^

**Figure 5:**
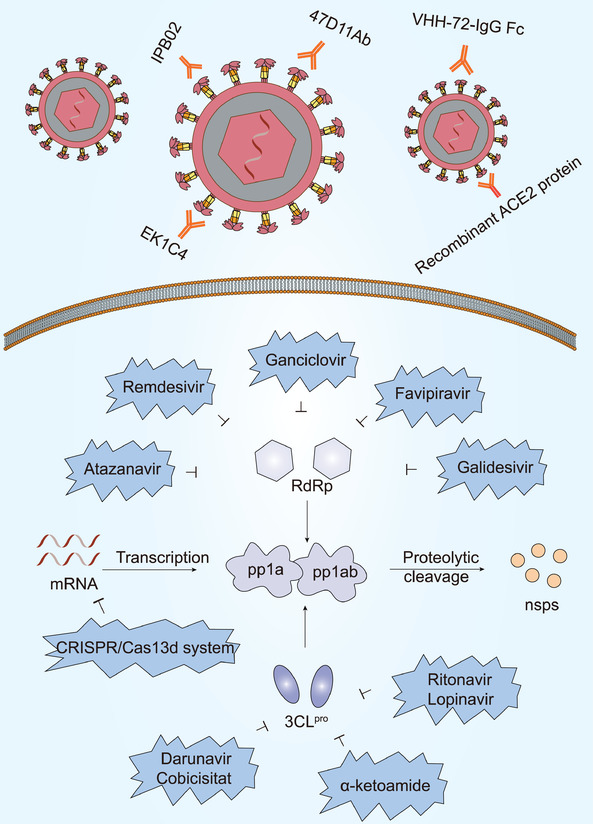
Virus‐based treatment approaches. EK1C4, 47D11 antibody, VHH‐72‐IgG Fc, IPB02, and recombinant ACE2 protein target S protein and inhibit SARS‐CoV‐2 infection. Atazanavir, Remdesivir, Ganciclovir, Galidesivir, and Favipiravir are shown to suppress RdRp function. Lopinavir, Ritonavir, Darunavir, Cobicistat, and *α*‐ketoamide are able to suppress 3CL^pro^ activity. CRISPR/Cas13d system targeting the viral genome has potential for inhibiting viral replication process.

Diverse proteins are essential for the survival of SARS‐CoV‐2, including 3CL^pro^, PL^pro^, and RNA‐dependent RNA polymerase (RdRp). It would therefore be wise to design drugs against these crucial viral proteins (**Figure** **5**). Previous studies have shown that ritonavir and lopinavir might have a therapeutic effect on SARS‐CoV‐2 infection for they could bind strongly to 3CL^pro^ and inhibit virus proliferation.^[^ ^98^, ^99^ ^]^ However, recent studies have suggested that treatment with lopinavir and ritonavir does not bring benefit to adults suffering from severe COVID‐19.^[^ ^100^ ^]^ Despite this, ten additional commercial drugs including colistin, valrubicin, lcatibant, bepotastine, epirubicin, epoprostenol, vapreotide, aprepitant, caspofungin, and perphenazine were predicted to interact with SARS‐CoV‐2 3CL^pro^ and have higher mutation tolerance by virtual screening.^[^ ^101^ ^]^ High‐throughput screening showed that four wildly used medicines (prulifloxacin, tegobuvir, bictegravir, and nelfinaviras) targeting SARS‐CoV 3CL^pro^ (≈96.1% sequence similarity to SARS‐CoV‐2 3CL^pro^) block the active sites or interrupts SARS‐CoV 3CL^pro^ dimer formation. Accordingly, this suggest that these same SARS‐CoV 3CL^pro^ ligands may also be useful for the treatment of SARS‐CoV‐2.^[^ ^102^ ^]^ Recently, an optimized *α*‐ketoamide, the inhibitor of 3CL^pro^, has been shown to exhibit pronounced lung tropism with almost no toxicity.^[^ ^103^ ^]^ Moreover, the newly designed peptidomimetic aldehydes 11a and 11b also displayed excellent anti‐SARS‐CoV‐2 3CL^pro^ activity with half‐maximal inhibitory concentrations (EC_50_) of 0.05 × 10^−6^ and 0.04 × 10^−6^ m, respectively, by occupying the substrate‐binding pocket of 3CL^pro^. Furthermore, these two ligands effectively inhibit SARS‐CoV‐2 infection in culture with half‐maximal effective concentrations (EC_50_) of 0.42 × 10^−6^ and 0.33 × 10^−6^ m, respectively. Both compounds showed good pharmacokinetic properties in vivo, and 11a also exhibited low toxicity.^[^ ^104^ ^]^

A drug‐target interaction model predicted that the anti‐HIV drug, Atazanavir, may bind to most subunits of the SARS‐CoV‐2 replication complex (i.e., RdRp, helicase, 3′‐to‐5′ exonuclease, 2′‐O‐ribose methyltransferase, and endoRNAse) and that Ganciclovir may bind to RdRp, 3′‐to‐5′ exonuclease, and RNA helicase. Furthermore, computational prediction also predicted that Darunavir may be a potential inhibitor of SARS‐CoV‐2 helicase, indicating that prezcobix, another anti‐HIV drug which contains darunavir and cobicistat, might be useful in treatment.^[^ ^105^ ^]^ However, whether drugs described above are able to function against SARS‐CoV‐2 remains unproven and clinical trials for these drugs need to be carried out before these drugs are used in the clinic.

As well as Atazanavir and Ganciclovir, numerous other antiviral medicines can serve as potential RNA polymerase inhibitors. Drugs such as Remdesivir and Galidesivir might be helpful against SARS‐CoV‐2 because they bind to RdRp.^[^ ^106^ ^]^ Further studies have indicated that Remdesivir might be a promising candidate because of its low‐micromolar EC_50_ (0.77 × 10^−6^ m), its high selectivity index (SI; >129.87), and its half‐cytotoxic concentration (CC_50_; >100 × 10^−6^ m).^[^ ^107^ ^]^ Interestingly, Remdesivir has already achieved good results in patients with SARS‐CoV‐2 infection.^[^ ^108^, ^109^ ^]^ Another RdRp‐targeting drug Favipiravir has been shown to reduce the incidence of fever and cough and promote the recovery of the patients. Thus, it is now recommended as a therapy for ordinary SARS‐CoV‐2‐infected patients without previous antiviral treatment.^[^ ^110^ ^]^

### 6.2 Virus‐Based Treatment Approaches: Antispike Protein Therapy

The SARS‐CoV‐2 S protein is also a critical drug target due to its unique role in viral survival (Figure 5). The SARS‐CoV‐2 S protein shares some homology with SARS‐CoV, which raises the interesting question of whether anti‐SARS‐CoV S protein antibodies share cross‐reactivity with the SARS‐CoV‐2 S protein. Anti‐SARS‐CoV antibodies mainly target the RBD (residues 318‐510) in the SARS‐CoV S protein.^[^ ^111^ ^]^ According to previous enzyme‐linked immunosorbent assay and the biolayer interferometry binding assay data, most of these antibodies (e.g., m396, CR3014, S230, and 80R) do not have affinity for SARS‐CoV‐2 RBD.^[^ ^13^, ^27^ ^]^ Although the SARS‐CoV‐specific human monoclonal antibody, CR3022, binds to the SARS‐CoV‐2 S protein, it does not neutralize SARS‐CoV‐2 in vitro even at high concentrations.^[^ ^27^, ^112^ ^]^ The polyclonal anti‐SARS S1 antibody, T62, does not inhibit entry of SARS‐CoV‐2 S pseudovirions.^[^ ^23^ ^]^ A monoclonal antibody 47D11, although unable to compromise spike‐receptor interactions, was shown to target the conserved core structure of the S1 RBD of SARS‐CoV‐2, thereby supporting viral clearance.^[^ ^13^ ^]^ Additionally, SARS‐CoV RBD‐directed single‐domain antibodies (VHHs) display cross‐reactivity against the SARS‐CoV‐2 RBD and can disrupt RBD dynamics and receptor‐binding but are unable to neutralize SARS‐CoV‐2 S VSV pseudoviruses. However, the VHH‐72‐IgG Fc fusion exhibited neutralizing activity against SARS‐CoV‐2 S pseudoviruses due to compensation of the high off‐rate constant.^[^ ^113^ ^]^

SARS‐CoV‐2 is capable of superior plasma membrane fusion compared to SARS‐CoV. Therefore, drugs that prevent membrane fusion may be useful in the treatment of SARS‐CoV‐2‐induced pneumonia. The lipopeptide, EK1C4, targets the HR1 domain and has been shown to inhibit SARS‐CoV‐2 S protein‐mediated cell–cell fusion and pseudovirus infection with IC_50_ values of 1.3 × 10^−9^ and 15.8 × 10^−9^ m, respectively. In addition, EK1C4 effectively inhibits the surface fusion of divergent CoVs (e.g., SARS‐CoV, MERS‐CoV, HCoV‐OC43, HCoV‐NL63, and HCoV‐229E). Therefore, EK1C4 might broadly inhibit S protein‐mediated membrane fusion of various *betacoronaviruses*.^[^ ^114^ ^]^ Moreover, peptides derived from the HR1 and HR2 sequences of S proteins have been shown to possess antiviral activity by binding to the S2 subunit of a prehairpin intermediate. The HR2 sequence‐based lipopeptide fusion inhibitor, IPB02, was shown to be a potential inhibitor of SARS‐CoV‐2 S protein‐mediated cell–cell fusion (IC_50_ = 0.025 × 10^−6^ m) and a potential inhibitor of SARS‐CoV‐2 pseudovirus infection (IC_50_ = 0.08 × 10^−6^ m).^[^ ^115^ ^]^

Given that ACE2 is a potent receptor for SARS‐CoV‐2, fusion proteins that contain the extracellular domain of ACE2 and Ig Fc domain may be able to target the virus, thereby inhibiting SARS‐CoV‐2 invasion of host cells.^[^ ^116^ ^]^ In vitro experiments have shown that both SARS‐CoV and SARS‐CoV‐2 are potently neutralized by ACE2‐Ig, which is composed of the extracellular domain of ACE2 and the Fc region of the human IgG1. Moreover, the neutralization effect remained when two histidine residues in the ACE2 active‐site were mutated to asparagine residues.^[^ ^117^ ^]^ The fusion proteins were long‐lasting in the plasma due to the recombinant Fc fusion technique; additionally, murine ACE2 fusion proteins induced few side‐effects in mice.^[^ ^118^ ^]^ Since ACE2‐Fc have potential to bind to SARS‐CoV‐2 and inhibit viral escape, such fusion proteins may be useful as prophylactic and therapeutic treatment of SARS‐CoV‐2. However, more research into this is desperately required.

### 6.3 Virus‐Based Treatment Approaches: Other Potent Therapies

The CRISPR/Cas13d system, specifically targeting the viral genome, may be useful in the prevention and treatment of SARS‐CoV‐2 and other RNA virus infections, with the aid of the adeno‐associated virus delivery system (Figure 5). Nonetheless, future studies are required to evaluate the safety and efficacy of this system as a potential antiviral therapy.^[^ ^119^ ^]^

The data‐driven drug repositioning framework designed by Ge et al. in 2020 revealed that the poly‐ADP‐ribose polymerase 1 (PARP1) inhibitor, CVL218, may serve as a potential therapeutic agent for SARS‐CoV‐2 treatment with no apparent toxicity.^[^ ^120^ ^]^ The results of that showed that CVL218 significantly inhibited viral multiplication, probably due to its interaction with the NTD of the SARS‐CoV‐2 N protein. Moreover, CVL218 was also shown to suppress CpG‐mediated IL6 production in PBMCs, thereby suggesting promising potential for the treatment of SARS‐CoV‐2‐induced pro‐inflammatory responses.

### 6.4 Host‐Based Treatment Approaches: Inhibition of Base Synthesis

Dihydroorotate dehydrogenase (DHODH) serves as a rate‐limiting enzyme in the fourth step of the de novo pyrimidine synthesis pathway and, significantly, DHODH inhibitors (e.g., teriflunomide) are thought to inhibit viral replication and eliminate cytokine storms. A recent study reported that teriflunomide might have the potential to treat SARS‐CoV‐2‐induced disease given that its EC_50_ and SI values are 6 µm and >33, respectively, at a multiplicity of infection (MOI) of 0.03. Additionally, compounds S312 (at MOI = 0.05, EC_50_ = 1.55 µm, SI > 64.62) and S416 (at MOI = 0.05, EC_50_ = 0.017 µm, SI > 5882.4), which were both identified by computer‐aided design, are also potent inhibitors of SARS‐CoV‐2.^[^ ^121^ ^]^ Equally, enzymes or genes that are involved in purine synthesis are also targets for SARS‐CoV‐2 drug discovery. RNA interference (RNAi) screening and CRISPR screening developed by Anderson et al. in 2020 recently identified the C‐1‐tetrahydrofolate synthase gene, MTHFD1, as a significant viral host factor. In vitro experiments have shown that carolacton, the MTHFD1 inhibitor, could potently block the replication of the Zika, mumps, and SARS‐CoV‐2 viruses with limited cytotoxicity.^[^ ^122^ ^]^ Therefore, carolacton may be a promising broad‐spectrum antiviral drug.

### 6.5 Host‐Based Treatment Approaches: Chloroquine and Hydroxychloroquine

**Figure 6:**
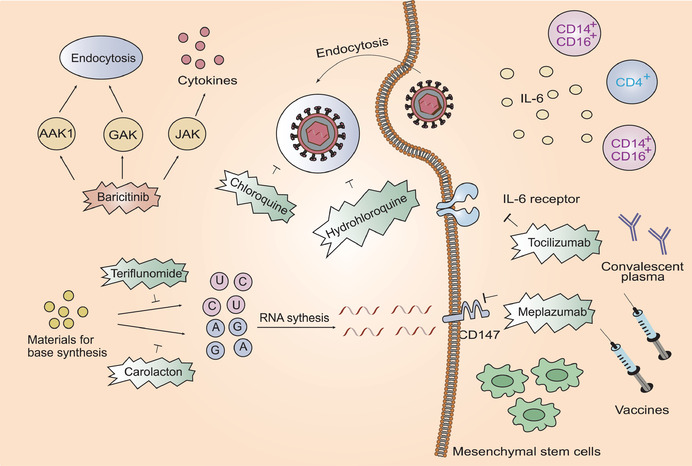
Host‐based treatment approaches. Chloroquine and hydrochloroquine suppress virus infection by increasing endosomal pH. Tocilizumab curbs immunopathology driven by SARS‐CoV‐2 via targeting IL‐6 receptor. Meplazumab targets a potential host receptor CD147. Baricitinib reduces inflammation and inhibits viral entry by binding to AAK1, JAK, and GAK. Teriflunomide interferes pyrimidine synthesis while Carolacton interferes purine synthesis. Mesenchymal stem cells are able to reduce cytokine storm. Convalescent plasma and vaccines are able to target virus and inhibit viral infection.

Previous studies have shown that the antimalarial drug, chloroquine, halts SARS‐CoV replication in the low micromolar range potentially via inhibition of membrane fusion events and disruption of ACE2 modifications.^[^ ^123^, ^124^ ^]^ Moreover, chloroquine is effective in the treatment of MERS‐CoV and HCoV‐229E, thereby suggesting its potential role as a broad‐spectrum antiviral agent against CoVs.^[^ ^123^ ^]^ A recent study demonstrates that chloroquine (EC_50_ = 0.77 × 10^−6^ m; CC_50_ > 100 × 10^−6^ m; SI > 129.87) stops SARS‐CoV‐2 infection and modulates antiviral immunity (**Figure** **6**).^[^ ^107^ ^]^ Additionally, hydroxychloroquine, an analog of chloroquine has been shown to reduce respiratory viral load and lessen the duration of SARS‐CoV‐2 carriage in most patients, with its impact reinforced by azithromycin.^[^ ^125^ ^]^ The efficacy of hydroxychloroquine was also elucidated in a randomized trial (Figure 6).^[^ ^126^ ^]^

### 6.6 Host‐Based Treatment Approaches: Anticytokine Storm Therapies

SARS‐CoV‐2 infection induces a cytokine storm that leads to deleterious clinical manifestations. Therefore, preventing the onset of a cytokine storm may be an effective option for the treatment of SARS‐CoV‐2‐induced pneumonia (Figure 6). The monoclonal antibody, tocilizumab, targets both membrane bound and soluble IL6 receptors and may potentially curb the immunopathology driven by SARS‐CoV‐2. Clinical trials aimed at verifying the efficacy of tocilizumab showed that it induced a decrease in body temperature and a swift improvement in the respiratory function of COVID‐19 patients.^[^ ^62^, ^127^ ^]^ Thus, tocilizumab may be of great benefit to patients infected with SARS‐CoV‐2 and it has already been proposed as one of the standard treatments for SARS‐CoV‐2 infection in the Diagnosis and Treatment Protocol for Novel Coronavirus Pneumonia (Trial Version 7) promulgated by the Chinese government.

CD147 has recently been identified as a potential host receptor for SARS‐CoV‐2.^[^ ^25^ ^]^ Previous studies also found that CD147 found in activated inflammatory cells participated in the modulation of cytokine induction and leukocytes chemotaxis by binding to cyclophilin A (CyPA).^[^ ^128^ ^]^ Therefore, the humanized IgG2 monoclonal antibody against CD147, meplazumab, is thought to be a promising therapeutic agent. The add‐on trials showed that compared to the control group, meplazumab accelerated viral clearance, promoted recovery, and suppressed inflammation in SARS‐CoV‐2‐infected patients.^[^ ^129^ ^]^ However, the efficacy and safety of meplazumab must first be elucidated in larger‐scale, randomized, and double‐blind clinical trials.

Baricitinib binds with high affinity to the adapter protein‐2 associated kinase 1 (AAK1), janus kinase (JAK), and the cyclin G‐associated kinase (GAK).^[^ ^130^ ^]^ Therefore, it may reduce inflammation and inhibit viral entry.^[^ ^131^ ^]^ Since the therapeutic dose of baricitinib (2 or 4 mg once daily) is ample to suppress AAK1, a clinical trial to evaluate the real‐world efficacy of baricitinib is necessary.

Mesenchymal stem cells (MSCs) are thought to modulate immune function and prevent cytokine storms.^[^ ^132^ ^]^ A report on seven patients who had received MSC transplants showed that after treatment, the functional outcomes of patients with severe disease were improved with the levels of lymphocytes increasing and the levels of the cytokine‐secreting immune cells decreasing.^[^ ^133^ ^]^ Besides, MSCs lack ACE2 and TMPRSS2, indicating that MSCs are unlikely to be targeted by SARS‐CoV‐2. Thus, the intravenous transplantation of MSCs may function as safe and effective treatment for infected patients, especially the severe cases.

### 6.7 Host‐Based Treatment Approaches: Corticosteroid

Corticosteroids may be useful for patients with hyperinflammation. Corticosteroids may reduce lung injury by inhibiting cytokine storms and promoting the absorption of exudative lesions. A recent report showed that among SARS‐CoV‐2‐infected patients who developed ARDS, treatment with methylprednisolone reduced the risk of death.^[^ ^134^ ^]^ Furthermore, the inhaled corticosteroid ciclesonide targets viral nsp15, thereby blocking CoV RNA replication.^[^ ^135^ ^]^ However, the use of corticosteroids to treat severe illness is controversial. Some clinical trials have shown that corticosteroids neither significantly accelerate patients improvement nor do they improve prognoses.^[^ ^44^ ^]^ Moreover, corticosteroids might delay viral clearance in the human body and exacerbate lung injury.^[^ ^131^, ^136^ ^]^ Hence, systematic corticosteroids treatment is not routinely recommended.

### 6.8 Host‐Based Treatment Approaches: Convalescent Plasma Therapy

Previous studies have shown that convalescent plasma reduces the mortality of patients infected with the causative agents of different viruses including severe influenza A, SARS‐CoV, and Ebola virus.^[^ ^136^, ^137^ ^]^ Thus far, there have been no effective and specific therapies against SARS‐CoV‐2 and hence, convalescent plasma therapy can serve as an exploratory attempt on the basis that most convalescent plasma contains neutralizing antibodies, which are theoretically capable of fighting against SARS‐CoV‐2 (Figure 6). A recent trial showed that this therapy could potentially improve clinical manifestations in severe patients infected with SARS‐CoV‐2.^[^ ^138^ ^]^ Of note, the collection of convalescent plasma must occur at the appropriate time to guarantee that it has a high neutralizing antibody titer.

### 6.9 Host‐Based Treatment Approaches: Vaccines

The CoV S protein not only binds to the receptors in the host via RBDs, but also mediates membrane confusion, which makes it a good target for the development of protective vaccines against SARS‐CoV‐2.^[^ ^139^ ^]^ Live‐attenuated vaccines, inactivated vaccines, the S gene DNA vaccines, and viral vectored vaccines have successfully vaccinated against the animal SARS‐CoVs.^[^ ^140^, ^141^ ^]^ Recent studies showed that an inactivated vaccine induced neutralizing antibodies against SARS‐CoV‐2 in mice, rats, and rhesus macaques,^[^ ^142^ ^]^ and DNA vaccine candidates encoding SARS‐CoV‐2 S protein could initiate humoral and cellular immune responses in rhesus macaques.^[^ ^143^ ^]^ Besides, some European countries have started studying whether the bacillus Calmette‐Guérin (BCG) vaccine can aid in the fight against SARS‐CoV‐2 by boosting the immune system.^[^ ^144^ ^]^

## 7 Conclusions and Perspectives

Through genomic and phylogenetic analyses, we have uncovered the genome sequences of SARS‐CoV‐2 and shown that this novel CoV is close to bat‐originated CoVs and pangolin‐CoV.^[^ ^145^ ^]^ Thus, bats may have been the original host of SARS‐CoV‐2 followed by subsequent transmission to humans by pangolins. However, the unique functions of most proteins encoded by the SARS‐CoV‐2 genome remain unknown. The majority of recent studies have mainly focused on the SARS‐CoV‐2 S protein that is the target of many vaccine development strategies and has been instrumental in aiding our understanding of the mechanism of viral invasion into host cells. Wrapp et al. in 2020 revealed the structural rearrangements of the S protein following entry of the virus into the host cells, and observed that SARS‐CoV‐2 showed 10–20‐fold increased affinity for ACE2 compared to SARS‐CoV. These data have served as important resources that have allowed us to make great progress in the study of this novel virus. However, some results are contradictory to other studies, which have shown that SARS‐CoV‐2 and SARS‐CoV have similar binding affinities to ACE2. Such discrepancies may be due to differences in experimental set ups or other unknown reasons, thereby necessitating further investigation. In addition, a 382‐nt deletion in the majority of the SARS‐CoV‐2 ORF8 gene was found in some genome sequences obtained from eight hospitalized patients in Singapore, which may be associated with reduced viral replicative fitness. Hence, this is likely to be an attenuated phenotype of SARS‐CoV‐2, which merits further investigation.

Through the comparison and analysis of numerous clinical cases, similarities in clinical manifestations have been observed, which will be helpful for future diagnosis and treatment of SARS‐CoV‐2. Although SARS‐CoV‐2 mainly causes respiratory symptoms, gastrointestinal symptoms such as diarrhea should not be neglected, as ACE2 is also highly expressed in the small intestines. Additionally, patients infected with SARS‐CoV‐2 can also be asymptomatic while still being able to infect other healthy individuals. Therefore, early identification of asymptomatic patients is essential.^[^ ^146^ ^]^ Chest CT scans are recommended for testing asymptomatic or high‐risk people who have ever exposed to SARS‐CoV‐2 infected patients in order to promote early identification of the disease.^[^ ^46^ ^]^ Cytokine storms play a crucial role in the development of SARS‐CoV‐ and SARS‐CoV‐2‐mediated pneumonia, especially in severe patients. Therefore, it is important that we study the dynamic changes in cytokines, chemokines, and immune cells of SARS‐CoV‐2‐infected people to further elucidate the mechanism(s) behind the immunopathology. Several studies have reported on the differences in cytokines between SARS‐CoV infection and SARS‐CoV‐2 infection. For instance, SARS‐CoV‐2 induces high IL10 expression while SARS‐CoV not. The mechanisms underlying such phenomena should be explored in more detail.

The development of treatments for CoVs is always a focus. In terms of high homology to other CoVs, the therapeutic methods applied for treating other viral causative agents may be useful for treating SARS‐CoV‐2‐infected patients. In essence, repurposing approved drugs is an efficient and cost‐effective approach. Nevertheless, the efficacy of most antiviral drugs predicted by various models needs to be tested for their real‐world efficacy and safety in clinical trials. Currently, several research groups are working on developing vaccines. The elucidation of the SARS‐CoV‐2 S protein structure and its interaction with ACE2 have significantly accelerated research into vaccine development. Nonetheless, the lengthy process of vaccine development often limits what clinicians can do to help patients suffering from acute disease. Therefore, it may be beneficial to isolate neutralizing antibodies from patients who have recovered from SARS‐CoV‐2. In conclusion, further research is required to improve our understanding of SARS‐CoV‐2, which will provide clues and pivotal data for diagnosis, prophylaxis, and treatment of the severe illness caused by SARS‐CoV‐2 and it is in urgent need to develop novel and efficacious broad‐spectrum drugs and vaccines against CoVs in order to find the best therapeutic agent to treat SARS‐CoV‐2 infections.

## Conflict of Interest

The authors declare no conflict of interest.

## Author Contributions

Y.D., T.D., and J.L. contributed equally to this work. Y.D. and T.D. conceived and drafted the manuscript. Y.D. drew the figures. J.L. discussed the concepts of the manuscript. F.Z. and L.Z. provided valuable discussion and revised the manuscript. The authors apologize to those researchers whose related work were not able to cite in this review.
